# Higher education as a platform for capacity building to address violence against women and promote gender equality: the Swedish example

**DOI:** 10.1186/s40985-018-0108-5

**Published:** 2018-11-19

**Authors:** Leah Okenwa-Emgwa, Eva von Strauss

**Affiliations:** 1 Department of Health Sciences, The Swedish Red Cross University College, Stockholm, Sweden; 2 Department of Occupational and Public Health Sciences, Faculty of Health and Occupational Sciences, University of Gävle, Gävle, Sweden

**Keywords:** Violence against women, Gender equality, Swedish higher education, European higher education, Quality assurance, Sweden, Public health workforce, Active citizens, women’s rights, Healthcare sector

## Abstract

Violence against women is an acknowledged public and global health problem which has adverse consequences for women’s health. Education, especially higher education, has long been identified as an important arena for addressing the problem and promoting gender equality. Two measures recently put in place in the Swedish higher education have brought the role of the sector into focus. The first is the inclusion of gender equality as a measurable outcome in quality assurance in higher education. The second measure is the amendment of the Swedish Higher Education Ordinance to include mandatory knowledge of VAW in the degree programme of seven selected relevant professional groups. The potentials of both measures to positively contribute to the gender equality discourse, as well as improving capacity building for the public health workforce who encounter VAW, are discussed.

## Background

There is an increasing focus on the role of the education sector as an important arena for addressing violence against women, VAW [1]. Due to the possibility of creating gender sensitive education and identifying at-risk females, schools, especially higher education, are considered potential agents of change. Two measures recently introduced within the Swedish higher education sector have brought the role of the sector into focus. First is the inclusion of gender equality (defined as equal rights, responsibilities and opportunities of women and men and girls and boys [2]), as a measurable outcome in quality assurance in higher education [3]. Second is the amendment of the Swedish Higher Education Ordinance to include mandatory knowledge of VAW in the degree programme description of professional groups likely to encounter victims and at-risk females in their line of duty [4]. This is part of a 10-year action plan to promote gender equality and address VAW [5].

VAW is a public health problem defined as “any act of gender-based violence that results in, or is likely to result in, physical, sexual, or psychological harm or suffering to women, including threats of such acts, coercion or arbitrary deprivation of liberty, whether occurring in public or private life” [6]. VAW could be physical, sexual or emotional and may occur as intimate partner violence (IPV), work place violence and harmful traditional practices such as female genital mutilation, dowry, forced marriage and honour-related killings [7]. Global lifetime prevalence of either physical and/or sexual intimate partner violence or sexual violence by a non-partner is about 35% [8]. As many as 82 % of female parliamentarians in a study in 39-country study across 5 regions, reported having experienced some form of threats, mobbing, remarks, gestures and images of a sexist or humiliating sexual nature against them [9]. Furthermore, about 1 in 7 girls were married or in union before age 15 [10] and up to 200 million women and girls have undergone female genital mutilation [11]. In Sweden, the Lifetime and past year prevalence of physical and/or sexual IPV are 28% and 5% respectively, while the lifetime non-partner sexual violence is 12% [12].

Consequences of VAW include injuries, mental health problems, homicide, sexually transmitted diseases and adverse reproductive health outcomes among others [8]. VAW transcends borders and is therefore a major global health problem. Forms of VAW, such as harmful traditional practices, common in Africa, the Middle East and South Asia [10, 11], are becoming common in places like Europe due to increasing migration. Furthermore, although VAW transcends age, the vulnerability of older women is often neglected in research and policies [13]. Few available studies show that VAW may be heightened for older women due to intensified inequalities, discrimination and human rights abuses associated with female ageing [13]. Recent projections show that the proportion of older women in populations is expected to increase globally by the year 2050 [14]. Given the above background, the need for increased knowledge and capacity building to address all forms of VAW cannot be overemphasised.

Research has shown that gender inequality is a significant driver of VAW [1, 5, 15–17]. Gender inequality is defined as “Legal, social and cultural situation in which sex and/or gender determine different rights and dignity for women and men, which are reflected in their unequal access to or enjoyment of rights, as well as the assumption of stereotyped social and cultural roles” [2]. Gender equality is a fundamental human right [1, 2], a social determinant of health [16] as well as a precondition for and indicator of sustainable development [2, 17]. According to the World Health Organisation (WHO), social construction of identity and unbalanced power relations between men and women, boys and girls often results in gender inequality which in turn often leads to poor health outcomes and decreased access to education for women in many contexts [16]. Efforts to promote gender equality do not mean that women and men will become the same, rather it is an attempt to create awareness that the rights, responsibility and opportunities of any individual should not depend on their gender.

Considering that one of the roles of higher education is to prepare students for life as active citizens [18], the sector can be considered as an important platform for promoting and highlighting the importance of gender equality. Sweden has over the years invested in harnessing the potentials of higher education to promote gender equality. Examples include projects to address gender dimensions and mechanisms related to students’ choice of programme of study, completion of studies or dropping out and pursuing doctoral level studies opportunities etc. [19]. The projects have also looked at gender issues regarding employment and leadership positions within universities and colleges [19]. Such projects have over the years informed policies promoting gender equality.

As mentioned earlier, one of the measures recently introduced into Swedish higher education is the inclusion of gender equality as a measurable quality assurance parameter. Higher education is an important social determinant of health and increases an individual’s employability and preparation for life as active citizens [1, 18]. A systematic process of quality assurance is thus necessary to ensure that students who pass through higher education acquire expected skills and knowledge. In the Swedish higher education, quality assurance is the responsibility of the Swedish Higher Education Authority (Universitetskanslersämbetet, UKÄ). Quality assurance is done via regular reviews and assessment carried out by an independent panel put together by UKÄ [3]. The panel often comprises of representatives from higher institutions, student unions, doctoral students, the labour sector, employee/employer organisations [3]. All members are nominated by their respective organisations and participate in the panel on equal terms [3].

The tool used for assessment is based on a model adapted to follow the standards and guidelines in the European higher education Area (ESG) [20]. The model has been developed to reflect relevant Swedish laws and ordnances and is still in its pilot phase [3]. It assesses seven key areas, as follows: governance and organisation, preconditions, design, implementation and outcomes, student and doctoral student perspective, working life and collaboration and finally gender equality [3]. The inclusion of gender equality in the Swedish assessment model means that gender equality must be taken into account, incorporated and adequately communicated in the design, content, implementation and delivery of all programmes within the Swedish higher education. Some concrete ways of doing this are, for example, conscious dialogue regarding gender equality when planning the programme structure; including gender equality as part of teaching-learning activities and ensuring a balanced gender representation in the selection of textbooks, teachers and supervisors [3].

The decision to include gender equality as a measurable outcome in the quality assurance process in higher education is laudable for many reasons. The inclusion contributes to achieving goal number five (i.e. gender equality) of the 17 sustainable development goals (SDGs) as well as all other SDGs closely related to number 5 [17]. Also, recent events have led to movements trying to highlight the problems of VAW. A good example is the current #MeToo wave against sexual harassment and assault that spread virally by social media in October 2017. The events pre and post #MeToo have led to discussions regarding what constitutes an acceptable behaviours and grey zones, e.g. differences between flirtations and harassment [21]. Much of the explanations for the cause of sexual harassment revolve around the problem of gender inequality, e.g. the degradation and objectification of women and power imbalance whether on the street, at school or at work, etc. [21]. Stakeholders have highlighted the need to reframe masculinity and redefine manhood as a step towards ensuring the respect and safety of females [21]. Incorporating gender equality in teaching, learning and administration within higher education will no doubt contribute to sensitising students (and also staff) on the importance of the subject. It will also help students to rethink norms and behaviours that have long been considered acceptable. The expected outcome is that in addition to acquiring skills relevant for employment, students will also be equipped for active citizenship in the discourse regarding gender equality, women’s rights and elimination of VAW.

The second measure introduced into the Swedish higher education is a targeted approach for capacity building to address VAW [4]. It involves an amendment of the Swedish Higher Education Ordinance to include knowledge about VAW in the education and training of seven selected relevant professional groups. These are professions likely to encounter groups vulnerable to VAW and victims. They include physiotherapy, law, medicine, nursing, social work, psychology and dentistry. From a health promotion perspective, education is an important strategy for the elimination of VAW, many stakeholders have long advocated its use [1]. Not much, however, has been done to actually equip professionals who regularly come in contact with victims of VAW. The inclusion of VAW in the programme description for these professions means that students must take mandatory courses on the subject during their training. This inclusion is thus a strategic move for many reasons.

One reason is that VAW is largely underreported and difficult to identify [22]. In clinical settings for example, three categories of victims (and potential victims) of VAW have been identified. They include those who disclose abuse or fear of it; those who do not disclose abuse, but present with abuse-related signs and symptoms such as bruises and reproductive health complications (e.g. lacerations and history of unexplained pregnancy complications); and lastly, those exposed to VAW but who neither show any signs of abuse nor report abuse [23]. Professionals therefore require adequate skills for screening, recognising warning signs and for the inter-sectorial collaboration needed for the care, safety and support of victims and vulnerable groups.

Another reason is that existing guidelines and standard routines for responding to VAW within the systems where these professionals operate (e.g. healthcare) are considered inadequate [24]. While the guidelines and standard routines serve as good frameworks for responding to VAW, they do not provide fundamental knowledge and competence needed to identify VAW and respond accordingly [25]. Interviews with victims of VAW have shown that a purely systems-based approach often lacks the type of response that is understanding, non-judgemental and sensitive to the complex nature of VAW [24]. It is therefore hoped that early exposure to the subject will provide professionals with a better understanding of the complexity of VAW and by proxy, a solid foundation upon which to effectively apply existing guidelines and standard routines. Proper response grounded in knowledge and understanding is a decisive factor for victims’ safety and further support.

Both measures are laudable and worth introducing in other higher education contexts and quality assurance models. A thorough approach is however needed to ensure their success. As part of the implementation process of the higher education quality assurance model, a series of trainings, guidelines, etc., have been put in place for reviewers, universities and colleges. These are part of concerted effort to ensure a smooth sail. For the second measure to be effective, a comprehensive mapping of specific knowledge and skills required by each of the seven professional group may be necessary. The foregoing is necessary in order to effectively plan the content and structure of VAW courses to be provided for each professional category. This is because, apart from general knowledge of VAW, skills required for screening, identification and handling of cases of VAW differ from one occupational group to the other. Conducting such mapping and planning at national level will also ensure a certain level of uniformity of what is delivered at universities and colleges across the country.

## Conclusions

In conclusion, these two measures despite being new and in their implementation phase [3, 4], have the potential to contribute immensely eliminating VAW in two specific ways. Firstly, by sensitising students about the importance of gender equality, they are prepared for active citizenship which is one of the goals of higher education [18]. This is specifically active citizenship in the form of contributing to the discourse and efforts to promote gender equality and eliminate VAW in their work and everyday life. Secondly, most of the professions among the selected degree programmes are part of the public health workforce. Introducing students to the issue of VAW early in their training means improved capacity building for the public health work force addressing VAW. If effectively executed, both measures have the potential to improve the health of women and populations. Results from future evaluation of both measures may provide further evidence base for the role of higher education in promoting gender equality and eliminating VAW.

## Abbreviations

ESG: Standards and Guidelines in European Higher Education Area
IPV: Intimate partner violence
SDGs: Sustainable Development Goals
UKÄ: Swedish Higher Education Authority (Universitetskanslersämbetet, UKÄ)
VAW: Violence against Women
